# Biocontrol agent *Bacillus amyloliquefaciens* LJ02 induces systemic resistance against cucurbits powdery mildew

**DOI:** 10.3389/fmicb.2015.00883

**Published:** 2015-08-28

**Authors:** Yunlong Li, Yilin Gu, Juan Li, Mingzhu Xu, Qing Wei, Yuanhong Wang

**Affiliations:** ^1^College of Horticulture and Landscape, Tianjin Agricultural University, Tianjin, China; ^2^State Key Laboratory of Microbial Resources, Institute of Microbiology, Chinese Academy of Sciences, Beijing, China

**Keywords:** powdery mildew, biocontrol agent, *Bacillus amyloliquefaciens*, salicylic acid, PR genes, systemic acquired resistance

## Abstract

Powdery mildew is a fungal disease found in a wide range of plants and can significantly reduce crop yields. Bacterial strain LJ02 is a biocontrol agent (BCA) isolated from a greenhouse in Tianjin, China. In combination of morphological, physiological, biochemical and phylogenetic analyses, strain LJ02 was classified as a new member of *Bacillus amyloliquefaciens*. Greenhouse trials showed that LJ02 fermentation broth (LJ02FB) can effectively diminish the occurrence of cucurbits powdery mildew. When treated with LJ02FB, cucumber seedlings produced significantly elevated production of superoxide dismutase, peroxidase, polyphenol oxidase and phenylalanine ammonia lyase as compared to that of the control. We further confirmed that the production of free salicylic acid (SA) and expression of one pathogenesis-related (PR) gene *PR-1* in cucumber leaves were markedly elevated after treating with LJ02FB, suggesting that SA-mediated defense response was stimulated. Moreover, LJ02FB-treated cucumber leaves could secrete resistance-related substances into rhizosphere that inhibit the germination of fungi spores and the growth of pathogens. Finally, we separated bacterium and its fermented substances to test their respective effects and found that both components have SA-inducing activity and bacterium plays major roles. Altogether, we identified a BCA against powdery mildew and its mode of action by inducing systemic resistance such as SA signaling pathway.

## Introduction

Cucurbits powdery mildew caused by *Sphaerotheca fuliginea* is a common disease of cucurbits under field and greenhouse conditions in most areas of the world (Perez-Garcia et al., 2009). Sulfur, copper, and various classes of fungicides or combinations of them are widely used to control those infections (Reuveni et al., 1996). Due to long-term extensive use of fungicides, pathogens have gradually evolved resistance to those fungicides. Moreover, the pesticide residues are of major concern because of their detrimental effects on human health and the environment (Zhang et al., 2015). To circumvent those undesirable effects, the application of biocontrol agent (BCA) is considered as a promising alternative treatment that can reduce both environmental pollution and the rise of fungicide resistance (Alamri et al., 2012).

Myriads of mechanisms have been attributed to BCA that keep plant from infections by pathogen (Haas and Defago, 2005). Different types of interaction mechanisms including: phytopathogen external competition, physical displacement of phytopathogens, secretion of anti-pathogen siderophores, synthesis of antibiotics and a variety of small molecules, production of enzymes that inhibit phytopathogens and induction of systemic resistance of the plant (Bargabus et al., 2004).

Generally, upon perception of specific compounds secreted by invasive pathogens, plants can initiate defensive mechanisms to counteract infections through a combination of constitutive as well as induced defenses such as systemic acquired resistance (SAR; Jung et al., 2005). SAR is effective against a wide range of pathogens and requires the synthesis of phenolic signaling compound, salicylic acid (SA; Feys and Parker, 2000). SAR is also known for the coordinate activation of a specific set of PATHOGENESIS-RELATED (PR) genes, several of which code for proteins with antimicrobial activities *in vitro* (Tornero et al., 1997). In addition, another different form of systemic resistance in plants responding to certain non-pathogenic rhizobacteria is referred to as induced systemic resistance (ISR) that is also effective against multiple pathogens (Feys and Parker, 2000). It is of note that ISR is independent of the SA production and PR induction but requires the operation of plant growth hormones jasmonic acid (JA) and ethylene signaling pathways. Although both SAR and ISR are effective against different types of pathogens, it was found that both SAR and ISR require *NPR1* gene in systemic plant defenses, suggesting the interplay of those systemic resistance (Feys and Parker, 2000).

*Bacillus amyloliquefaciens* was separated from *Bacillus subtilis* as a new species (Priest et al., 1987) and both have been reported as BCAs in controlling cucumber powdery mildew (Chen et al., 2013). As spore-forming bacteria, *B. amyloliquefaciens* possesses several advantages and make them good candidates of BCA. Previous studies showed that the crude protein of antifungal agents could inhibit conidial production effectively (Li et al., 2009). Besides, *B. amyloliquefaciens* produces spores with strong resistance to adverse conditions that provide convenience for commercial uses (Arguelles-Arias et al., 2009). Importantly, their antagonistic effect is mainly dependent on the production of antibiotics and siderophores. *B. amyloliquefaciens* produces various antibacterial and antifungal antibiotics such as surfactin, iturin, and fengycin (Chen et al., 2009). Both iturins and fengycins are recently shown to have major roles in antagonism toward *Podosphaera fusca* infecting melon leaves (Romero et al., 2007).

In this study, we isolated a BCA named LJ02 from greenhouse soil against cucumber powder mildew and characterized it as *B. amyloliquefaciens* using combinatorial analyses. In order to elucidate the protective mechanisms of LJ02, the activity of resistance-related enzymes, the production of SA and the expression of *PR-1* gene in cucumber leaves were monitored after treating plant leaves with 1% LJ02 fermentation broth (LJ02FB). Moreover, we examined the inhibitory ability of LJ02-induced root secretions against pathogen (*Fusarium oxysporum*, *Botrytis cinerea*, and *Alternaria* spp).

## Materials and Methods

### Isolation of LJ02 and Source of Pathogen Fungi

The LJ02 strain was isolated from greenhouse soil in Tianjin, China. The soil samples were air-dried and sifted through 60 mesh sieve. Five g soil sample was suspended in 45 mL of 0.9% NaCl on a shaker at 200 rpm for 30 min. The suspension was diluted 10^3^ ∼ 10^4^ times in 0.9% NaCl. 100 μL of the suspension was spread onto sterile LB plates (For 1,000 mL, tryptone: 10 g, yeast extract: 5 g, NaCl: 10 g, agar: 12 g, distilled water: 1,000 mL, pH 7.2 ∼ 7.4) and incubated at 28°C for 48 h. Single colonies were selected and re-streaked for pure culture. The pure cultures were stored in 25% glycerol solution at –80°C. LJ02 and pathogen fungi (*S. fuliginea*, *F. oxysporum*, *B. cinerea* and *Alternaria* spp.) were stored in Laboratory of Plant Protection, Tianjin Agriculture University.

### Plant Growth Conditions

Cucumber seeds (*Cucumis sativus* cv *Corona*, Jinchun 4) were obtained from Tianjin Kernel Cucumber Institute. The seeds were surface-sterilized for 10 min in 30% sodium hypochlorite and rinsed three times with distilled water. The seeds were then incubated at 28°C for 24 h on the sterile wet filter paper. Then the sprouted seeds were planted in the mixture of peat soil and vermiculite in 9-cm pots and the seedlings were used for greenhouse trials, detection of resistance-related enzymes, SA production, detecting of fungi quantity and monitoring the expression of *PR-1* gene. As for the inhibition effects on pathogenic fungi, the seedlings were planted in 250 mL erlenmeyer flask containing 20 mL MS solid medium (For 1,000 mL, KNO_3_: 1.9 g, MgSO_4_·7H_2_O: 0.37 g, NH_4_NO_3_: 1.65 g, KH_2_PO_4_: 0.17 g, MnSO_4_·4H_2_O: 29 mg, ZnSO_4_·7H_2_O: 8.6 mg, H_3_BO_3_: 6.2 mg, CoCl_2_·6H_2_O: 25 μg, Na_2_MoO_4_·2H_2_O: 250 μg, CuSO_4_·5H_2_O: 25 μg, CaCl_2_·2H_2_O: 44 mg, KI: 830 μg, glycine: 2 mg, aneurine hydrochloride: 0.4 mg, pyridoxine hydrochloride: 0.5 mg, nicotinic acid: 0.5 mg, Na_2_-EDTA: 37.25 mg, FeSO_4_·7H_2_O: 27.85 mg, inositol: 0.1 g, agar: 7 g, distilled water: 1000 mL, pH 6.0; Murashige and Skoog, 1962). The MS liquid medium was used to detect the germination rate. All seedlings were cultivated in growth chamber with 16 h day (10,000 lx, 25°C) and 8 h night (20°C) at 60% relative humidity.

### Preparation of LJ02FB and Greenhouse Trial

LJ02FB was prepared by the following methods. Strain LJ02 was inoculated into 5 mL of LB liquid medium and cultured to stationary phase. Then 100 μL of LJ02 LB culture was inoculated into 100 mL PDB medium (For 1,000 mL, potato: 200 g, glucose: 20 g, distilled water: 1,000 mL) and cultivated at 28°C for 40 h at 200 rpm. Then LJ02FB [10^9^ CFU (colony forming unit)/mL] was diluted 100 times (v/v) into sterilized water (1% LJ02FB). Fresh powdery mildew (*S. fuliginea,* Verhaar et al., 1996) were obtained from infected cucumber leaves in Laboratory of Plant Protection, Tianjin agriculture university. The spores were brushed in sterile water for spore suspension and the concentration was adjusted to 10^6^ CFU/mL. The concentration was determined by counting spores using a Neubauer hemocytometer (Xu et al., 2006). To test the efficacy of strain LJ02 against cucurbits powdery mildew, each cucumber seedling was firstly sprayed with pathogen spores during cotyledon period. As soon as disease spots were observed, the seedlings of treatment group were sprayed with 1% LJ02FB (∼10^7^ CFU/mL, 5 mL). 1% PDB medium (5 mL) was used for control group. Ten seedlings were used as one group for greenhouse experiments and each trial was repeated at least three times. The efficacy on disease severity was investigated for 3 weeks. The disease severity were scored using a modified rating (*r*) standard, denoting proportions of disease over the whole leaf area, where: 0: 0%, 1: <1%, 3: 2 ∼ 5%, 5: 6 ∼ 20%, 7: 21 ∼ 40%, and 9: >40% (Yan et al., 2006). Then, the disease severity index (DI) and control efficacy (CE) was computed using the formula as follows (Yan et al., 2006):

DI (%) = [Σ(*rn_r_*)/9*N_t_*] × 100

Where *r* is rating value, *n_r_* is number of diseased leaves with a rating of *r*, and *N_t_* is total number of leaves tested.

CE (%) = [1-(*ck*/*t*)] × 100

Where *ck* is DI of control group, *t* is DI of treated group.

### PCR Amplification and Sequencing of *gyrB* Gene

Total DNA of strain LJ02 was extracted by using Solarbio DNA kit (Solarbio). Primer pair: up1 (5′-GAAGTCATCATGACCGTTCTGCAYGCNGGNGGNAARTTYGA-3′) and up2r (5′-AGCAGGGTACGGATGTGCGAGCCRTCNACRTCNGCRTCNGTCAT-3′) were used to amplify the *gyrB* gene (Yamamoto and Harayama, 1995). Amplified fragment were purified by DNA gel purification kit (Omega Bio-Tek) according to the manufacturer’s instructions and then sequenced by Shanghai Sangon Biological Engineering Technology and Service Co. Ltd., China. The *gyrB* gene sequence was compared with GenBank database by the BLAST program and the phylogenetic tree was constructed using the neighbor-joining method with the MEGA 5.1 program (Tamura et al., 2011).

### Physiological and Biochemical Analysis

Gram staining and spore staining were observed through microscope. Physiological and biochemical properties of strain LJ02 were identified according to Bergey’s Manual of Systematic Bacteriology (Vos et al., 2009).

### Detection of Activity of Resistance Related Enzymes

The cucumber seeds were cultured in MS solid medium as described above (see *Plant growth conditions*). Each cucumber seedling was sprayed with 1% LJ02FB (5 mL) at the three-leaf stage. 1% PDB liquid medium (5 mL) was applied as a control. Each treatment was repeated three times in all groups. Cucumber leaves were randomly collected at 0.5, 1, 3, 5, 7 and 11 days after treatment to detect the activity of superoxide dismutase (SOD), peroxidase (POD), polyphenol oxidase (PPO), and phenylalanine ammonia lyase (PAL). 0.4 g leaves were ground in liquid nitrogen and homogenized at 4°C in 2 mL of 0.05 mol/L phosphate buffer (pH 6.8). Homogenate (crude enzyme solution) was centrifuged at 4°C at 12,000 rpm for 20 min. The supernatant was used for further analyses.

#### Detection of SOD Activity

Superoxide dismutase activity was determined using a NBT method as described previously with some modifications (Pokora et al., 2003). 0.05 mL of enzyme liquid was added to the reaction mixture. The tubes without addition of enzyme solution (replaced by phosphate buffer) were taken as control. The SOD activity was measured by optical density (OD) at 560 nm.

#### Detection of POD Activity

Peroxidase activity was determined as described with modifications (Wang et al., 2012). The mixture was reacted in 2.65 mL of 0.05 mol/L phosphate buffer (pH 6.8). And the condition of bathing was changed into 30°C for 5 min before adding hydrogen peroxide. Control groups were performed in the absence of enzyme liquid. The OD_470_ value was measured for 5 min, and one unit of enzyme activity was defined by the change in absorbance of 0.1 per minute.

#### Detection of PPO Activity

The activity of PPO was determined according to the method with some modifications (Esterbauer et al., 1977). The reaction mixture contained 0.10 mL enzyme liquid and tubes without addition of enzyme solution were taken as control. Then OD_495_ value of reaction liquid was measured for 5 min, and one unit of enzyme activity was defined by the change in absorbance of 0.1 per minute.

#### Detection of PAL Activity

Leaves (0.4 g) were ground in liquid nitrogen and homogenized at 4°C in 2 mL of 0.05 mol/L borate buffer (pH 8.8, containing 5 mmol/L mercaptoethanol and 1 mmol/L EDTA). Homogenate was centrifuged at 4°C at 10,000 rpm for 15 min. The supernatant was used for analyses. The activity of PAL was determined as described with some modifications (Li et al., 2008). The reaction mixture contained 3.8 mL of borate buffer, 1.0 mL of 0.02 mol/L L-phenylalanine and 0.2 mL enzyme liquid. The tubes without addition of enzyme solution were taken as a control. Then, all the tubes were bath at 40°C and the OD_290_ value was determined per 15 min until it kept steady, and one unit of enzyme activity was defined by the change in absorbance of 0.1 per hour.

### Detection of SA

The cucumber seedlings were grown in the conditions as described above (see *Plant growth conditions*). Two types of treatment were carried out as follows.

#### LJ02FB Induced SA Production

Each cucumber seedling was sprayed with 1% LJ02FB (5 mL) at three-leaf stage. 1% PDB medium (5 mL) was sprayed as a control. Cucumber leaves (2 g) were randomly collected at 0.5, 1, 3, 5, 7, and 11 days after treatment to detect the free and conjugated SA.

#### Bacterium and its Fermented Substances Induced SA Production

The LJ02FB culture was precipitated and bacterial pellet was suspended in 10 mM MgCl_2_ solution (LJ02BC), and its fermented substances were filter sterilized (LJ02FS). The cotyledons of cucumber seedlings was sprayed with LJ02BC (2 mL) and LJ02FS (2 mL) separately in two groups at three-leaf stage. 1% PDB medium (2 mL) was sprayed in control groups. The upper uninoculated leaves (2 g) of cucumber seedlings and the roots (including main and lateral roots, 2 g) were collected at 0.5, 1, 3, 5, 7, and 11 days after treatment to detect the free SA.

Salicylic acid fractions including free SA and conjugated SA were extracted as described with minor modifications (Palva et al., 1994). The extracts were dissolved in 500 μL methanol and spotted on silica gel plates. Then the plates were developed in a solvent system consisting of petroleum ether (60–90): *n*-hexane: ethyl acetate: acetic acid at the volume ratio of 10:30:15:1. The SA was detected by observing a UV reflected band with an Rf value corresponding to that of the standard SA. The samples were scraped from silica gel plates and dissolved in 1 mL methanol. The suspension was centrifuged at 12, 000 rpm for 5 min and filtered through a 0.22 μm filter and stored at –20°C.

Detection of SA samples was performed using a Shimadzu LC-20AT HPLC equipped with a UV-detector. 20 μL crude extraction of SA was injected into a C-18 reverse-phase column (diameter × length: 4.6 × 150 mm) at 25°C. SA was separated with 80% methanol (v/v) in 0.1% acetic acid solution with a flow rate of 0.8 mL/min and detected under the wavelength of 300 nm.

### Detection of *PR-1* Gene Expression

To evaluate the expression of *PR-1* gene in cucumber, each cucumber leave was sprayed with 1% LJ02FB (2 mL) at three-leaf stage. 1% PDB was sprayed as control. The cucumber leaves were sampled 0.5, 1, 2, and 3 days after treatment. The samples were ground by mortar and pestle in liquid nitrogen. Total RNA was extracted using the Total RNA Isolation Kit (Solarbio). One microgram of total RNA was converted into cDNA using Reverse Transcription Reagent Kit (Takara) according to the instructions. The primers for qPCR were used as follows: PR1-forward 5′-TGCTCAACAATATGCGAACC-3′ and PR1-reverse 5′-TCATCCACCCACAACTGA AC-3′ (Alizadeh et al., 2013); 18S-forward 5′-TCTGCCCGTTGCTCTGATG-3′ and 18S-reverse 5′-TCACCCGTCAC CACCATAG-3′ (Wan et al., 2010). The length of amplified fragments were between 100 and 200 bp. PCR reaction mixture (20 μL) consisted of 10 μL SYBR *Premix Ex Taq* (Tli RNaseH Plus), 2 μL of each forward and reverse primer. 2 μL cDNA, 4 μL dH_2_O. Then real-time PCR was performed in CFX96 Real-Time PCR Detection System (Bio-Rad) with the following parameters: 95°C for 30 s followed by 40 cycles at 95°C for 3 s and 60°C for 30 s. Gene *18S* was used as the reference gene of cucumber. Relative gene expression was calculated by the Bio-Rad CFX Manager 2.1 software.

### Germination Rate of Pathogenic Spore

The exudate of cucumber rhizosphere was collected by the following method. The seedlings were cultured in sterile MS solid medium and then were transplanted into a flask containing 20 mL sterile MS liquid medium when the first leaf sprouted. All seedlings were incubated at 25°C in sterile conditions with illumination intensity of 10,000 lx and illumination time of 16 h/day period. After 2 days, leaves including cotyledon were spotted with 20 μL of the LJ02FB (∼10^9^ CFU/mL) as treatment group. 20 μL of the sterile PDB medium was spotted in control group. After 2 days, 30 μL of the MS liquid medium from LJ02FB-treated group was mixed with 30 μL of pathogen spore suspension (*F. oxysporum*, *B. cinerea*, and *Alternaria* spp., 150 ∼ 200 spores per sample) on the concave glass. Each treatment was repeated three times and the concave glass were incubated at 28°C. The germination of spores was recorded after incubation for 2 ∼ 9 days, respectively.

### Detection of Fungi Quantity in the Cucumber Rhizosphere

Counting the fungi in cucumber rhizosphere treated by LJ02FB was conducted by the following steps. The cucumber seedlings were cultured in seedling-raising dish as described above. 1% and 10% LJ02FB were sprayed with 20 μL per leaf at trefoil stage. The CFU of each fungus in the cucumber rhizosphere was determined as described previously (Faheem et al., 2015). Five cucumber plants were pulled out of each group and the soil was shook off and blended from the roots. The soil suspension was prepared by adding one gram of the soil into 9 ml of sterile distilled water and mixing for 10 min. Serial dilutions were subsequently prepared in sterile distilled water. 100 μL of the suspension was spread on selective Martin medium (For 1,000 mL, glucose: 10 g, tryptone: 5 g, K_2_HPO_4_: 1 g, MgSO_4_·7H_2_O: 0.5 g, agar: 15 g, 1% Rose Bangal: 0.33 mL, 1% streptomycin: 3 mL, distilled water: 1,000 mL; Xu and Zheng, 1986) and incubated at 25°C. The number of CFU was recorded on each plate after 3 days. All experiments had at least five replicates.

### Inhibition of LJ02-Induced Root Secretions Against Pathogen Fungi

The inhibition against pathogen fungi of LJ02-induced secretions was detected as described previously with minor modifications (On et al., 2015). Briefly, 20 μL of the LJ02FB was dropped onto the leaves including cotyledon under sterile condition at trefoil stage. After treated for 48 h, cucumber seedlings were pulled from the MS solid medium. The MS solid medium containing rhizosphere secretions was made into cake by a sterile puncher (0.5 cm in diameter). Each pathogen fungus (*F. oxysporum*, *B. cinerea,* and *Alternaria* spp.) was also made into fungi cake by a sterile puncher (0.5 cm in diameter) which was then inoculated onto the center of PDA plate. Four pieces of MS solid medium cake were inoculated around the fungi cake at a distance of 2.5 cm (treatment group). The cake made from sterile MS solid medium without root secretions was used as a control. Each treatment was repeated three times and cultured at 28°C for 9 days. Subsequently, the inhibition zone diameter was detected.

### Statistical Analysis

The data of disease index, CE, resistance enzyme activities, SA production, germination rate, and fungi pathogen counts in rhizospheres were analyzed by one-way ANOVA. Duncan’s multiple-range test was applied when one-way ANOVA revealed significant differences (*P* < 0.05). All statistical analysis was performed with SPSS version 11.5 statistical software (SPSS, Chicago, IL, USA).

## Results

### LJ02 is a New Member of *B. amyloliquefaciens*

One-hundred-fifteen isolations were collected to test their antagonistic activity according to their inhibitory activity. One strain was selected and named LJ02. Greenhouse trials showed the CE on cucurbits powdery mildew of ∼70, ∼90, and ∼85% after treated with 1% LJ02FB for 7, 14, and 21 days, respectively (Table 1). The results proved that strain LJ02 indeed inhibited the occurrence of cucurbits powdery mildew under greenhouse condition. Therefore, we intended to identify the LJ02 based on its phylogeny, morphology, physiological, and biochemical properties.

**TABLE 1:** **Disease index and control efficacy of powdery mildew in cucumber**.

**Treatment**	**7 days after treatment**		**14 days after treatment**		**21 days after treatment**	
	DI (%)	CE (%)	DI (%)	CE (%)	DI (%)	CE (%)
1% LJ02FB	3.67 ± 0.55 a	69.80	5.93 ± 0.59 a	86.44	7.80 ± 0.3 a	85.05
Control	12.17 ± 1.01 b	–	43.87 ± 2.76 b	–	53.60 ± 10.34 b	–

Data expressed as the mean ± SD. Different letters indicate significant difference at P < 0.05 according to Duncan’s multiple range test.

**FIGURE 1:**
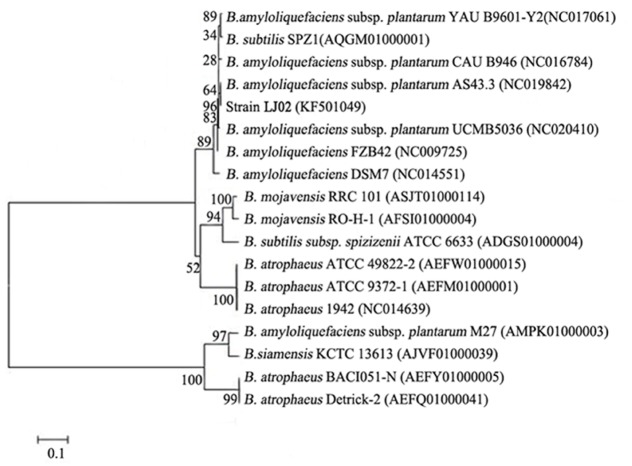
**Phylogenetic tree of strain LJ02 and other ***Bacillus*** species from GenBank based on ***gyrB*** gene sequences.** The tree was constructed by MEGA5.1 by the neighbor-joining method. Genetic distances were computed by Kimura 2-parameter model. The *gyrB* gene sequence of LJ02 was submitted to GenBank database and the accession number is KF501049.

Phylogenetic analysis of LJ02 was determined using *gyrB* gene as a reference (Yamamoto and Harayama, 1995). An 1.2 kb *gyrB* gene fragment was amplified from the strain LJ02 and sequenced. The *gyrB* gene sequence of LJ02 was aligned with published sequences of related taxa obtained from GenBank. Phylogenetic tree was therefore constructed and demonstrated high homology to *B. amyloliquefaciens* (Figure 1).

**FIGURE 2:**
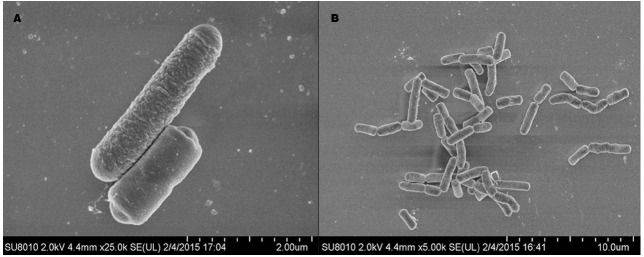
**Scanning microscope observation of LJ02 under two resolutions: 25,000 × (A) and 5,000 × (B).** The images were taken from a ultra-high resolution scanning electron microscope SU8010.

Strain LJ02 colonies show light yellow on LB agar medium. Gram staining and microscopic observation showed that LJ02 is Gram-positive, endospore-forming and rod-shaped bacterium with a width of 0.7–0.9 μm and a length of 1.8–3.0 μm, indicative of a typical bacillus morphology (Figure 2).

The physiological and biochemical properties were determined and shown in Table 2. Specifically, strain LJ02 is aerobic and cannot grow under anaerobic conditions. Strain LJ02 produces H_2_S and can use citrate as sole carbon source. It is also positive for catalase, nitrate reduction, and Voges–Proskauer test and negative for oxidase, indole production and egg yolk reaction. LJ02 produces acid from xylose, sorbitol, *L*-arabinose, starch, inositol, sucrose, glucose, galactose, ribose, glycogen, and glycerol. All features are highly identical to those of *B. amyloliquefaciens* described in Manual of Systematic Bacteriology (Vos et al., 2009).

**TABLE 2:** **Physiological and biochemical characteristics of strain LJ02**.

**Physiological and biochemical index**	**Strain LJ02**	**Physiological and biochemical index**	**Strain LJ02**
Catalase activity	+	Indole production	–
Nitrate reduction	+	Growth with 0.001% lysozyme	+
H2S produced	+	Egg yolk reaction	–
Utilization of citrate	+	Anaerobic growth	–
Arginine dihydrolase	–	β-Galactosidase	–
Lysine decarboxylase	–	Oxidase activity	–
Ornithine decarboxylase	–	Methyl red test	–
V. P. Test	+	Temperature for growth range	15–50 C
Phenylalanine deaminase	–	Motility	+
Growth in NaCl range at (%, w/v)	0–10%	Litmus milk test	+
**Hydrolysis of**		**Utilization of sole carbon source**	
Starch	+	Starch	+
Gelatin	+	Inositol	+
Aesculin	+	*L*-Arabinose	+
Casein	+	*D*-Trehalose	+
Tyrosine	–	Glycerol	+
Urea	–	Ascorbic acid	–
Tween 20	+	Proline	+
Tween 80	–	Cystine	–
**Acid produced from**		Threonine	–
Xylose	+	Valine	–
Rhamnose	+	Arginine	–
Sorbitol	+	Citric acid	–
Mannitol	+	Sucrose	+
*L*-Arabinose	+	Xylose	+
Starch	+	Maltose	+
*D*-Trehalose	+	Tyrosine	–
Inositol	+	Mannitol	+
Sucrose	+	Glucose	+
Fructose	+	Sorbitol	+
Maltose	+	Fructose	+
Glucose	+	Oxalate	–
Mannose	+	Galactose	+
Galactose	+	Ribose	+
Ribose	+	Glycogen	+
Glycogen	+	Rhamnose	–
Glycerol	+	Mannose	–

“+” means positive; “–” means negative.

Altogether, the isolated BCA was referred to as *B. amyloliquefaciens* LJ02 based on its morphological, phylogenetic, physiological, and biochemical characterizations.

### LJ02FB Induces the Production of Resistance-Related Enzymes in Cucumber Leaves

During initial screening of LJ02, greenhouse trials indicated that LJ02 is effective in controlling cucurbits powdery mildew over 20 days. In view of this long-term effect, we hypothesized that the systemic induced resistance (SIR) may take place when LJ02 was applied. To this end, the activity of systemic resistance-related enzymes was firstly examined.

**FIGURE 3:**
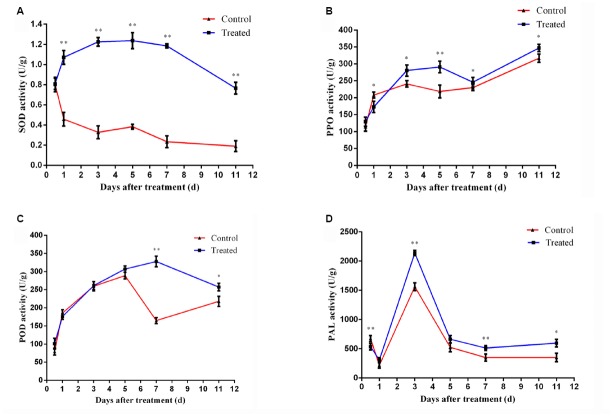
**Time course of changes in SOD activity (A), PPO activity (B), POD activity (C), and PAL activity (D) in cucumber leaves of control and treatment group.** Error bars indicate standard deviation among triplicates. A one-way ANOVA was performed (*P < 0.05; **P < 0.01).

As is shown in Figure 3A, the treatment group had a significant increase in SOD production as compared to the control treatment. The SOD activity of treatment group increased gradually and peaked after 36 h. It is very interesting to notice that the SOD activity retained a higher level even after 11 days treatment, suggesting its major role in disease resistance under our conditions.

As is shown in Figure 3B, there was slight difference between the control and treatment group plants at the primary treatment period (<2 days). But the activity of PPO increased rapidly after 2 days and became significant different (*P* < 0.05) from the control at 3rd day.

The rapid increase of POD activity was observed during the first 3 days after treatments and the difference between treatment and control became evident from 7th day (Figure 3C). This is probably because of the production of excess H_2_O_2_ by the action of increased SOD in LJ02FB treated plants.

Both group induced a rapid increase of PAL activity levels in cucumber leaves within 1–3 days. The PAL activities of either group reached the top level at 3rd day, while treatment group induced significantly stronger PAL activity (*P* < 0.05) than the control at the maximum inducing time point (Figure 3D).

All those results confirmed that LJ02FB treatment could indeed induce the production of SIR-related enzymes.

### LJ02FB Enhances Production of SA in Cucumber Leaves

**FIGURE 4:**
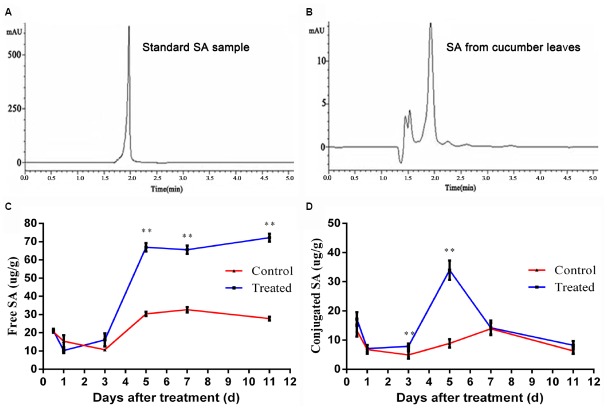
**HPLC chromatogram of SA standard (A) and sample (B), temporal changes of free SA (C) and conjugated SA (D) in cucumber leaves of control and treatment group.** Error bars indicate standard deviation among triplicates. A one-way ANOVA was performed (*P < 0.05; **P < 0.01).

Salicylic acid is an important plant-produced signaling molecule involved in SAR (Park et al., 2007). It is responsible for inducing tolerance to a number of biotic and abiotic stresses, which is also a trigger for the production of SAR-related enzymes (Korkmaz et al., 2007). Thus, we established a method to detect the concentration of SA in LJ02FB-treated cucumber leaves (Figures 4A,B). We extracted the free SA and the sugar-conjugated SA from LJ02FB-treated plant leaves and monitored the trend of SA accumulation in the cucumber leaves in time-course experiments. We found that the accumulation of free SA on cucumber leaves increased rapidly after 5-day treatment as compared to control (Figure 4C). In addition, we also observed an increase of conjugated SA (day 3 ∼ 5) with a subsequent decline in production from LJ02FB-treated leaves (Figure 4D). Thus, we come to the conclusion that functional SA production was enhanced in cucumber leaves to stimulate SA-mediated defense response, such as the expression of resistance-related enzymes.

### Expression of SA-Dependent *PR-1* Gene is Induced in LJ02FB-Treated Cucumber Leaves

**FIGURE 5:**
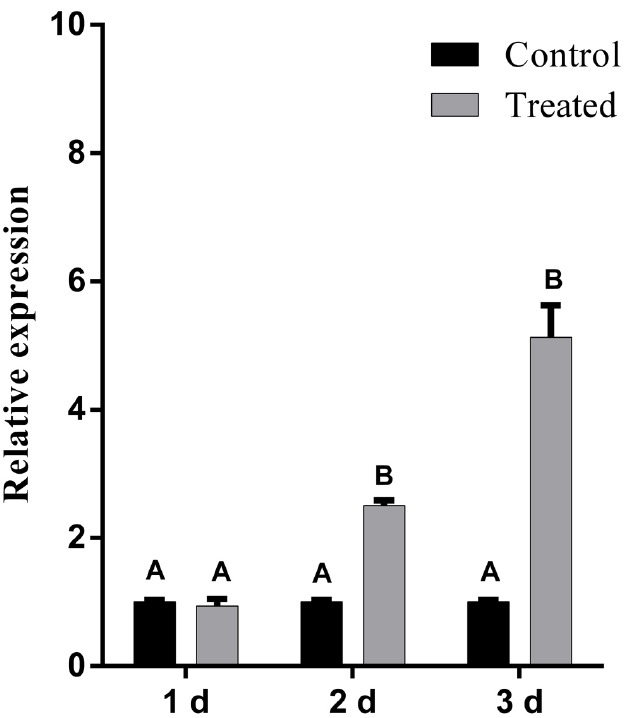
**Relative expression of ***PR-1*** gene in cucumber leaves after treated with LJ02FB (Treated) and PDB (Control).** Error bars indicate standard deviation among triplicates. Different letters are significantly difference at *P* < 0.01 according to Duncan multiple range test.

The enhanced production of total SA in cucumber leaves led us to put forward the hypothesis that defense-related genes are probably induced. To this end, we analyzed the level of transcription of *PR-1* gene, a commonly used maker for SA-mediated expression (Tornero et al., 1997). In order to investigate the effect of LJ02FB on this process, the expression of *PR-1* in cucumber leaves was studied after treated with LJ02FB. As is shown in Figure 5, at 2nd day and 3rd day after treated with LJ02FB, the expression of *PR-1* gene in treatment group was significantly higher than that of control group, indicating that SA-mediated defense gene expression is induced in LJ02FB-treated cucumber leaves.

### LJ02FB Treatment Elicits a Long-Range Resistance Against Fungi in Cucumber Rhizosphere

**FIGURE 6:**
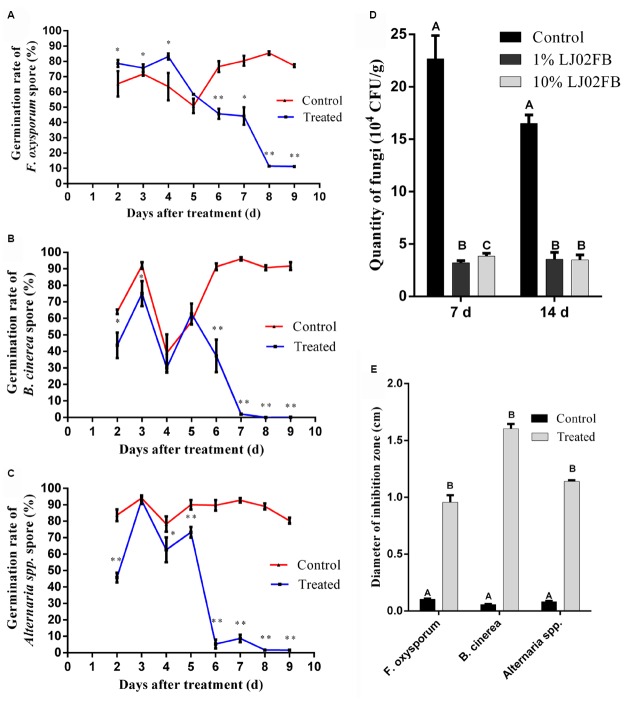
**Long-range resistance against fungi in cucumber rhizosphere.** Germination rate of *F. oxysporum* **(A)**, *B. cinerea* **(B)**, and *Alternaria* spp. **(C)** spores with different treatments. Error bars indicate standard deviation among triplicates. A one-way ANOVA was performed (*P < 0.05; **P < 0.01). **(D)** The quantity of pathogenic fungi in the rhizosphere of cucumber treated by different concentrations of LJ02FB in leaves. Error bars indicate standard deviation among triplicates. Different letters are significantly difference at *P* < 0.01 according to Duncan multiple range test. **(E)** The comparison of the inhibition zone of cucumber rhizosphere MS solid medium against different pathogenic fungi (cm). Error bars indicate standard deviation among triplicates. Different letters are significantly difference at *P* < 0.01 according to Duncan multiple range test.

In order to further unravel the protective mechanisms of LJ02FB on disease control, the rhizosphere exudates of LJ02FB-treated cucumber were co-cultured with three common pathogen spores. As is described previously, *S. fuliginea* is an obligate parasite which is hard to culture on nutrient medium (Perez-Garcia et al., 2009). Therefore, we detected the diameter of inhibition zone against other pathogen fungi (*F. oxysporum*, *B. cinerea,* and *Alternaria* spp.) through plate cultivation test. Most spores would germinate at 9 h in the preliminary period. However, with the accumulation of antagonistic substance, the germination of all pathogens spores began to decrease significantly at 5th day and reached the lowest level at 8 ∼ 9 days. The final germination rate is about 10% for *F. oxysporum* (Figure 6A), less than 1% for *B. cinerea* (Figure 6B) and less than 2% for *Alternaria* spp. (Figure 6C).

Moreover, the quantity of rhizosphere fungi was determined after treated with LJ02FB in cucumber leaves. As is shown in Figure 6D, after treated with LJ02FB in cucumber leaves, the number of rhizosphere fungi declined significantly. In line with 7th day, the quantity of fungi at 14th day is still significantly less than that of control group (*P* < 0.01). Based on this findings, we further revealed that the rhizosphere MS solid medium of LJ02FB-treated cucumber could strongly inhibit the growth of *F. oxysporum*, *B. cinerea*, and *Alternaria* spp. (Figure 6E).

All those results showed that LJ02FB could elicit the defense responses from plant leaves to rhizosphere, suggesting a long-range systemic resistance is induced under our test conditions. Besides, the examination of rhizosphere secretions in solid MS medium was proved to be an effective method that identifies potential BCAs with resistance-induction and anti-fungal activities.

### Both LJ02 Bacterial Cells and Their Fermented Substances Induce SA Production in Systemic Tissues

To further dissect the functions played by bacterium and its fermented substances of LJ02FB, we separated them by centrifugation and kept bacterial cells in 10 mM MgCl_2_ solution (LJ02BC) and filtered the supernatants to rule out the contamination of bacterial cells to harvest fermented substances (LJ02FS). We inoculated both fractions onto cucumber cotyledons to treat plants and then extracted the SA samples from upper uninoculated true leaves and in roots after 0.5, 1, 3, 5, 7, and 11 days. In doing so, we could examine the effects of both fractions (LJ02BC and LJ02FS) on the production of SA in cucumber tissues with spatial and temporal scales.

**FIGURE 7:**
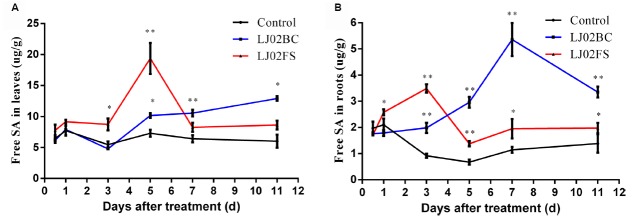
**Spatial and temporal detection of SA with separated LJ02FB components (LJ02BC and LJ02FS).** Time course of changes in free SA activity in leaves **(A)** and in roots **(B)** of control and treatment group. Error bars indicate standard deviation among triplicates. A one-way ANOVA was performed (*P < 0.05; **P < 0.01).

As can be seen from Figure 7A, the accumulation of free SA in upper uninoculated cucumber leaves increased rapidly after 3-day treatment as compared to control. Both LJ02BC and LJ02FS could induce the production of free SA in upper leaves throughout all experiments. This confirmed our speculation that LJ02 could stimulate the SA-mediated SAR response in cucumbers. Interestingly, LJ02FS could induce significantly higher amount of free SA at 5th day and declined to control level. This is possibly caused by the degradation of functional elicitors in LJ02FS. However, we observed that LJ02BC could consistently induce ∼twofold increase in free SA production, suggesting that microbe-associated molecular pattern (MAMP) may be responsible for SA stimulation (Newman et al., 2013).

In addition, we also detected an increase of free SA (day 3 ∼ 11) in cucumber roots with both LJ02BC- and LJ02FS-treatment (Figure 7B). And it appeared that LJ02BC played a major role in this process, although the amount of free SA was lower as compared to that of leaves. This phenomenon led us to conclude that LJ02 could induce a long-range of defense response by promoting the free SA production, which will then elicit the SA signaling pathways to enhance the immunity of cucumber plants systemically.

## Discussion

The search for the BCA against powdery mildew has been a long standing practice for plant pathologists. In this study, we isolated a BCA against powdery mildew and identified it as a new member of *B. amyloliquefaciens*. Interestingly, we further found a large body of evidences that LJ02 could induce SA-mediated SAR as one of its major mode of actions. To be specific, when treated with LJ02FB, cucumber seedlings produced significantly high amount of SOD, POD, PPO, and PAL. The production of free SA and the expression of SA-dependent *PR-1* gene in cucumber leaves were also enhanced markedly after treating with LJ02FB. Moreover, we determined that TJ02FB-treated cucumber leaves secreted resistance-related substance into rhizosphere against a range of fungi pathogens, suggesting that LJ02 could elicit a long-range systemic resistance in cucumber against pathogens. Finally, we further dissect the roles played by LJ02BC and L02FS and found that LJ02 could really elicit SAR response in leaves as well as in roots. Overall, our work provides insights into SA-mediated defense response as a mode of action by *B. amyloliquefaciens* LJ02 against powdery mildew. Furthermore, we have proved that LJ02-induced long-range resistance is an appropriate indicator to examine the biocontrol ability, especially the SAR-inducing activity, of different BCAs using solid rhizosphere medium. Therefore, the established detection method could further promotes the identification of BCAs against agricultural infections or pathogens.

At the first beginning, the identification of LJ02 as *B. amyloliquefaciens* is different from previous studies since we could not distinguish it from *B. subtilis* based on 16S rRNA comparison (data not shown). Therefore, we switched to the other method described by Yamamoto using *gyrB* as a marker to perform phylogenetic analysis (Yamamoto and Harayama, 1995). Although 16S rRNA sequence has been commonly used as a simple method for the identification of microorganisms, it has limitations for constructing bacterial phylogenetic relationships due to the high percentage of sequence similarity between closely related species. At present, *gyrA*, *gyrB*, *rpoB*, and *rpoD* gene sequences have been used for identifying the *Bacillus* sp. and *Pseudomonas* sp. except 16S rRNA gene sequence (Yamamoto and Harayama, 1995). And it was shown that *gyrB* gene sequences provide higher resolution than 16S rRNA gene sequences (Wang et al., 2007). Therefore, it is likely that using *gyrB* as a phylogenetic marker is reliable in our study and could provide clues for bacillus determination.

The main mechanisms of BCAs include the production of antibiotics, competition, plant growth promotion, and the induction of SAR and ISR (Ramarathnam et al., 2011). Generally, it was believed that beneficial bacteria can make the plant more tolerant to pathogens by stimulating ISR, which was shown to protect above-ground plant tissues and acts through roots to leaves (Van der Ent et al., 2009). Typically, SAR can perceive the invasion of pathogens by initiate defensive responses via the synthesis of SA and the coordinate activation of a large set of PR proteins and resistance-related enzymes (Feys and Parker, 2000). It was also reported that enhanced resistance against phytopathogens by exogenous elicitor application is also associated with defense-related enzymes (Song et al., 2011).

In our study, the activities of resistance-related enzymes including SOD, POD, PPO, and PAL considerably increased after treated by LJ02FB. Interestingly, as an major defensive enzyme, SOD showed significantly elevated activity. The main function of SOD is eliminating the cellular superoxide radical (O_2_^–^), and the increase of SOD activity can lead to the accumulation of H_2_O_2_. It is proved that H_2_O_2_ appears to be a key element involved in disease resistance to pathogens (Ahmad et al., 2010). Therefore, BCA-induced production of SOD could provide an extra protection against pathogen infection of plants. However, the excess production of H_2_O_2_ will lead to the production of POD that scavenge H_2_O_2_ (Yamasaki et al., 1997). Additionally, we also found that the activity of PPO was also stimulated during LJ02 treatment. PPOs function as catalyzing the oxygen-dependent oxidation of phenols to quinones and are assumed to be involved in plant defense against pests and pathogens (Li and Steffens, 2002). It was shown that overexpression of PPO in transgenic tomato plants can lead to enhanced disease resistance (Li and Steffens, 2002). PAL was also enhanced during LJ02 treatment. It was shown to catalyze the deamination of L-phenylalanine to produce cinnamic acid, a substrate feeding into several biosynthetic routes to various classes of phenylpropanoid-derived secondary plant products, including SA (Mauch-Mani and Slusarenko, 1996).

The content of free SA is known to increase in dozens of times when plant tissues are affected by incompatible pathogens and elicitors (Leon et al., 1995). And there is compelling evidences that SA acts as a signal molecule in plant disease resistance (Vlot et al., 2009) and the *PR* gene family act as reliable markers of SA-mediated response or SAR (Hunt and Ryals, 1996). Intriguingly, in our study, we detected a significant increase in the content of free SA in cucumber leaves during LJ02 treatment. This is very intriguing since we used the fermentation broth of a beneficial bacterium rather than a pathogen to treat plant leaves. Besides, we found that the production of several resistance-related enzymes has been significantly stimulated upon LJ02 treatment, which was probably caused by the coordinate activation by enhanced SA levels in LJ02-treated plants. Actually, it was reported that PAL and PPO are the main SAR-related enzymes in plants (Jung et al., 2011). As we know, only free SA is involved in the signal transduction in plant disease resistance (Park et al., 2007). Our work demonstrated that the content of free SA in cucumber leaves was kept at a relatively higher level after spraying the LJ02FB. Previous experiments had the same results after they inoculated the pathogens on various plants (Verberne et al., 2000). Furthermore, the expression of *PR-1* gene was detected by qRT-PCR. We found that *PR-1* gene was stimulated after treated with LJ02FB in cucumber leaves at different time points. Among PR genes, *PR-1* expression is a paradigm for the co-regulation of *PR* genes and correlates well with the induction of SAR (Durrant and Dong, 2004).

To further unravel the long-range effect of LJ02FB treatment, we tested the biocontrol activity of root exudates or secretions against common pathogenic fungi. We found extremely evident induced defensive response of root exudates against different pathogens including *F. oxysporum*, *B. cinerea* and *Alternaria* spp. Considerable decrease in spore germination and fungal growth were detected after treated with LJ02FB. This data further confirmed that LJ02 could induce a systemic resistance response against pathogens, perhaps through secreting anti-fungal substances into rhizosphere, possibly through leave-to-root translocation or local synthesis of those substances. This further promoted us to note that LJ02 could induce a long-range defense response from leaves to roots in unknown mechanisms. Subsequently, we found that when we separated the LJ02FB into LJ02BC and LJ02FS, LJ02BC played very important roles in inducing the production of free SA in both upper uninoculated leaves and in roots. Moreover, LJ02FS also has SA-inducing activity with relative lower level as compared to that of LJ02BC, indicating both fractions are important for SA-inducing activity. Those results led us to conclude that SAR is indeed induced by LJ02 and MAMP is likely to be responsible for the triggering of SA-dependent defense such as SA synthesis and the subsequent pathogen inhibition (Porcel et al., 2014). Besides, the long-range elicitation of SA induction in roots may be involved in aforementioned long-range resistance.

Beneficial bacteria and pathogens are initially recognized as harmful invaders in order to limit their spreading (Trda et al., 2015). Plants commonly use plasma-membrane localized pattern recognition receptors (PRRs) to perceive MAMPs/pathogen-associated molecular patterns (PAMPs; Newman et al., 2013). These are conserved signatures of crucial microbial structures, such as cell wall components or motility organs (Boller and Felix, 2009) as well as cyclic lipopeptides found in *B. subtilis* (Farace et al., 2015). PRR-mediated microbe sensing gives rise to a wide array of defense responses known as MAMP- or PAMP-triggered immunity (MTI/PTI; Zipfel, 2014). MTI is a defense program with complex early signaling events including ion fluxes, mitogen-activated protein (MAP) kinase cascade activation and the production of reactive oxygen species (ROS; Garcia-Brugger et al., 2006). SA as a key immune signal is involved in the regulation of downstream defense genes (Robert-Seilaniantz et al., 2011). Also the SA accumulation triggered by MAMPs is a major component of the MAMP-triggered signaling mechanism (Tsuda et al., 2008).

As can be seen from our results (Figure 7) that MAMP associated with LJ02 is perhaps the underlying mechanism for the SA accumulation in cucumber tissues since we could detect the increase of SA production when treated with LJ02BC. The LJ02FS is also functional to stimulate the SA production both in upper leaves and roots but not as potent as that of LJ02BC. This is likely to be caused by the degradation of potential elicitors in fermentation broth. *B. subtilis* is known to produce cyclic lipopeptides such as surfactins, iturins and fengycins (Farace et al., 2015). It was reported that purified surfactin, mycosubtilin, and plipastatin could be perceived by grapevine plant cells to stimulate grapevine innate immune responses including SA-mediated pathways (Farace et al., 2015). Therefore, it is conceivably that similar substances will be produced in its close species, *B. amyloliquefaciens* and similar immune responses will be induced. However, the different effects of LJ02BC and LJ02FS on the SA-mediated defense responses may attributed to certain unidentified MAMP factors that functions in association with bacterium itself.

Another interesting finding in our study is that the SA signaling pathways induced by beneficial bacterium such as LJ02 is likely to function from leaves (aboveground, AG) to roots (belowground, BG; Figure 7B). Although we did not detect a large amount of SA induced in roots, it is clearly that LJ02 could significant induce its production in a temporal manner. Therefore, LJ02 could employ MAMP to stimulate SA accumulation in both AG (SAR) and BG (SA signaling pathway) and in doing so to exert its biocontrol activities against a wide range of foliar and rhizospheric pathogens. In previous studies, only one-way signal transduction was investigated, such as AG–BG, AG–AG, and BG–BG (Yi et al., 2011). Recently, the bidirectional signal exchanges between AG–BG was identified as another type of induced resistance response. It was reported that foliar attack by the whitefly not only elicited AG resistance against a leaf pathogen, *Xanthomonas axonopodis* pv. *vesicatoria*, but also elevated resistance against the soil bacterium, *Ralstonia solanacearum*. And another interesting finding of this study is that AG whitefly feeding significantly increased the population density of beneficial BG microflora including actinomycetes and saprophytic fungi that may induce systemic resistance (Yang et al., 2011). Very recently, it was discovered that SA could modulate colonization of the root microbiome by specific bacterial taxa, indicating that SA is likely to be important for the colonization or recruitment of beneficial microbes in rhizosphere (Lebeis et al., 2015).

**FIGURE 8:**
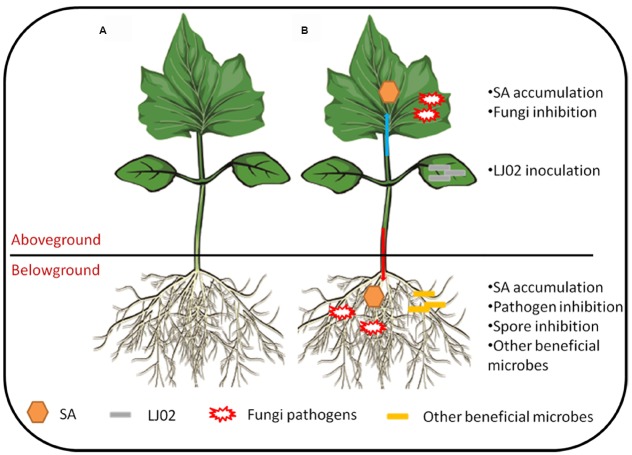
**Hypothetical model of LJ02-mediated protection of cucumber plants. (A)** A cucumber seedling under normal condition; **(B)** LJ02 inoculation elicits SA-dependent signaling pathway in both directions: AG–AG and AG–BG. For more details, please refer to main text.

Based on current data and those previous studies, we summarized our results and some hypotheses in Figure 8. It can be seen that upon inoculation of LJ02 and in AG part, the upper cucumber leaves synthesized elevated level of SA and at the same time, this could partially explain the inhibition of powdery mildews. In BG part, the long-range resistance responses lead to the inhibition of several pathogens and their spore germination. Besides, the free SA was also increased in roots and it is possible that some beneficial bacteria could also be recruited to rhizospheres to protect plants from pathogenic invasions.

Based on the long-range resistance responses, we have established a method that could easily detect the inhibitory effect induced by foliar BCAs. We proved that induced rhizosphere secretions in solid MS medium by treating plant leaves with BCAs was an effective method to examine the anti-fungal and long-range resistance activities of potential BCAs. Although it is similar to that described for disk diffusion method (On et al., 2015), our new method takes the advantage of the coordinate activation of anti-fungal substances by plant-BCA-pathogen interactions rather than traditional BCA-pathogen interactions. The new approach will broaden our understanding of protective mechanisms by potential BCAs and can be applied to other beneficial microbes.

### Conflict of Interest Statement

The authors declare that the research was conducted in the absence of any commercial or financial relationships that could be construed as a potential conflict of interest.
